# Immuno-priming durvalumab with bevacizumab in HER2-negative advanced breast cancer: a pilot clinical trial

**DOI:** 10.1186/s13058-020-01362-y

**Published:** 2020-11-11

**Authors:** Miguel Quintela-Fandino, Esther Holgado, Luis Manso, Serafin Morales, Begoña Bermejo, Ramon Colomer, Juan V. Apala, Raquel Blanco, Manuel Muñoz, Eduardo Caleiras, Vega Iranzo, Mario Martinez, Orlando Dominguez, Javier Hornedo, Lucia Gonzalez-Cortijo, Javier Cortes, Ariadna Gasol Cudos, Diego Malon, Antonio Lopez-Alonso, María C. Moreno-Ortíz, Silvana Mouron, Santos Mañes

**Affiliations:** 1 Breast Cancer Clinical Research Unit – Clinical Research Program, CNIO - Spanish National Cancer Research Center, Melchor Fernandez Almagro, 3, 28029 Madrid, Spain; 2 Medical Oncology Department, Hospital Universitario de Fuenlabrada, Fuenlabrada, Spain; 3 Medical Oncology Department, Hospital Universitario Quiron, Pozuelo de Alarcon, Spain; 4 Medical Oncology Department, Hospital Universitario Ramon y Cajal, Madrid, Spain; 5 Medical Oncology Department, Hospital Universitario 12 de Octubre, Madrid, Spain; 6 Medical Oncology Department, Hospital Universitari Arnau Vilanova, Lleida, Spain; 7 Medical Oncology Department, Hospital Clínico Universitario, Valencia, Spain; 8 INCLIVA, Valencia, Spain; 9 CIBERONC, Instituto Carlos III, Madrid, Spain; 10 Medical Oncology Department, Hospital Universitario La Princesa, Madrid, Spain; 11 Facultad de Medicina, Universidad Autónoma de Madrid, Madrid, Spain; 12 Department of Immunology and Oncology, Centro Nacional de Biotecnología/CSIC, Darwin, 3, 28049 Madrid, Spain; 13 Histopathology Core Unit – Biotechnology Program, CNIO - Spanish National Cancer Research Center, Madrid, Spain; 14 Medical Oncology Department, Hospital General Universitario de Valencia, Valencia, Spain; 15 Medicine Department, Universitat de Valencia, Valencia, Spain; 16 Pathology Department, Hospital Universitario 12 de Octubre, Madrid, Spain; 17 Genomics Core Unit – Biotechnology Program, CNIO - Spanish National Cancer Research Center, Madrid, Spain; 18 ION Institute of Oncology, Quironsalud Group – Madrid & Barcelona, Barcelona, Spain; 19 Vall d’Hebron Institute of Oncology, Barcelona, Spain

**Keywords:** Durvalumab, Bevacizumab, HER2-negative breast cancer, Vascular normalization, Immuno-priming

## Abstract

**Background:**

Preclinical research suggests that the efficacy of immune checkpoint inhibitors in breast cancer can be enhanced by combining them with antiangiogenics, particularly in a sequential fashion. We sought to explore the efficacy and biomarkers of combining the anti-PD-L1 durvalumab plus the antiangiogenic bevacizumab after bevacizumab monotherapy for advanced HER2-negative breast cancer.

**Methods:**

Patients had advanced HER2-negative disease that progressed while receiving single-agent bevacizumab maintenance as a part of a previous chemotherapy plus bevacizumab regimen. Treatment consisted of bi-weekly durvalumab plus bevacizumab (10 mg/kg each i.v.). Peripheral-blood mononuclear cells (PBMCs) were obtained before the first durvalumab dose and every 4 weeks and immunophenotyped by flow-cytometry. A fresh pre-durvalumab tumor biopsy was obtained; gene-expression studies and immunohistochemical staining to assess vascular normalization and characterize the immune infiltrate were conducted. Patients were classified as “non-progressors” if they had clinical benefit (SD/PR/CR) at 4 months. The co-primary endpoints were the changes in the percentage T cell subpopulations in PBMCs in progressors versus non-progressors, and PFS/OS time.

**Results:**

Twenty-six patients were accrued. Median PFS and OS were 3.5 and 11 months; a trend for a longer OS was detected for the hormone-positive subset (19.8 versus 7.4 months in triple-negatives; *P* = 0.11). Clinical benefit rate at 2 and 4 months was 60% and 44%, respectively, without significant differences between hormone-positive and triple-negative (*P* = 0.73). Non-progressors’ tumors displayed vascular normalization features as a result of previous bevacizumab, compared with generally abnormal patterns observed in progressors. Non-progressors also showed increased T-effector and T-memory signatures and decreased T_REG_ signatures in gene expression studies in baseline—post-bevacizumab—tumors compared with progressors. Notably, analysis of PBMC populations before durvalumab treatment was concordant with the findings in tumor samples and showed a decreased percentage of circulating T_REGs_ in non-progressors.

**Conclusions:**

This study reporting on sequential bevacizumab+durvalumab in breast cancer showed encouraging activity in a heavily pre-treated cohort. The correlative studies agree with the preclinical rationale supporting an immunopriming effect exerted by antiangiogenic treatment, probably by reducing T_REGs_ cells both systemically and in tumor tissue. The magnitude of this benefit should be addressed in a randomized setting.

**Trial registration:**

**(**www.clinicaltrials.gov): NCT02802098. Registered on June 16, 2020.

## Background

Single-agent immune checkpoint inhibitors have shown moderate efficacy in advanced breast cancer [1–5]. Considerable efforts are directed towards enhancing their activity, most often in combination with chemotherapy, but also in chemotherapy-free regimens [6].

Several factors modulate the response of cancer cells to anti-PD-1/PD-L1 agents, including tumor mutational burden (TMB), PD-L1 expression, and immune infiltration [7, 8]. PD-L1 expression rates [9, 10] are usually low in breast cancer. Several strategies have been suggested to enhance the efficacy of immunotherapy, including the manipulation of tumor microenvironment to enhance T effector cell infiltration and access to the tumor [11]. Recently, it has been suggested that combining antiangiogenic therapies and immunotherapies might increase the effectiveness of immunotherapy [12].

VEGF-A (Vascular Endothelial Growth Factor-A), the main soluble factor responsible for tumor angiogenesis, is also involved in creating an immunosuppressive tumor microenvironment: it prevents dendritic cell (DC) precursors from evolving into mature antigen-presenting DCs and promotes the development of tumor-associated macrophages [13, 14]. It also fosters the proliferation of immunosuppressive cells, limits T cell recruitment into tumors, and promotes T cell exhaustion [15]. Tumor vessel and stromal abnormality correction (which can be achieved by antiangiogenics) can restore the immune function [16]. Preclinical evidence in breast, pancreatic, and brain tumors and melanoma [17, 18] and clinical results in renal cell cancer [19] have shown that antiangiogenic treatment was followed by more efficient T cell extravasation and tumor infiltration, turning immunologically ‘cold’ into ‘hot’ tumors [17, 18]. Thus, combining antiangiogenics with immune checkpoint inhibitors represents a potentially interesting chemo-free regimen. Several recent trials combining small-molecule antiangiogenics plus immune checkpoint inhibitors have shown enhanced activity particularly in settings where high VEGF levels are known to drive tumor growth [20]. The role of VEGF as a driver of breast cancer progression, however, has been extensively questioned in light of the long-term results of phase III trials with antiangiogenics [21–24].

The efforts invested in studying immune-boosting combinations, however, have paid little attention to the timing of administration of the “immune enhancer”. The natural rhythms of innate and adaptive response could be used to enhance the efficacy of anti-PD-1/PD-L1 agents by combining them with agents in the right sequence [25, 26]. In that sense, it is possible that pre-treatment with a VEGF-A inhibitor could exert an immuno-priming effect that would increase the chance of response to subsequent immune checkpoint blockade and be more effective than concurrent therapy [26], while causing less adverse events [12]. We sought to explore a sequential chemotherapy-free regimen combining the anti-PD-L1 agent durvalumab with the antiangiogenic agent bevacizumab. In order to take advantage of the potential “sequential-effects”, patients had to have experienced disease progression while on bevacizumab monotherapy prior to entering the trial (as maintenance treatment after a chemotherapy plus bevacizumab regimen). In addition, we sought to find traits in peripheral-blood mononuclear cells (PBMCs) and tumor biopsies that informed about the immune priming, in an attempt to search for novel biomarkers and obtain a better understanding of the biology of this clinical scenario.

## Methods

We performed a prospective, open-label, multicentric, single-arm, phase IB investigator-initiated study. The study was conducted in accordance with the Declaration of Helsinki and Good Clinical Practice standards and registered at Clinicaltrials.gov (NCT02802098). Ethics approval was obtained from the Madrid Regional Government Ethics Board for Drug Research and the Spanish Agency for Medicine and Health Products (AEMPS).

### Study population

Women ≥ 18 years old were eligible if they had a non-curable, locally advanced or metastatic HER2-negative histologically confirmed breast cancer. Given the academic nature of this study, and the limited funding as a result of being an independent study, in the context of current FDA status for bevacizumab, the only option to execute a pilot trial in order to detect a signal of “priming effect” was to nest a state-of-the-art set of correlative studies in patients that were still receiving bevacizumab as a part of previously FDA-approved bevacizumab+chemotherapy combination regimens followed by bevacizumab maintenance. A clinical trial where candidates were started on bevacizumab monotherapy was deemed unrealistic and possibly unethical by the investigators, and thus, the key inclusion criterion was that it was mandatory to have been receiving bevacizumab monotherapy maintenance for a minimum of 6 weeks as a part of any bevacizumab-containing regimen prior to registration, but there was no limit to the number of previous treatment lines. The only restriction was that no previous bevacizumab-containing regimens were allowed (i.e., it had to be the first bevacizumab-containing regimen that the patients were exposed to). Other inclusion criteria included negative pregnancy test, ECOG (Eastern Cooperative Oncology Group) 0–1, adequate organic function (according to standard definitions), lack of uncontrolled brain metastases, LVEF (Left Ventricular Ejection Fraction) > 45%, and life expectancy > 24 weeks. Patients suffering from concurrent severe conditions, taking anticoagulants, receiving immunosuppressants, or with a history of clinically significant bleeding or thromboembolic events within 6 months or study entry were excluded. Previous treatment with immunotherapy was not allowed. Non-measurable disease (e.g., bone disease only) was allowed.

### Trial design and correlative studies

**Fig. 1:**
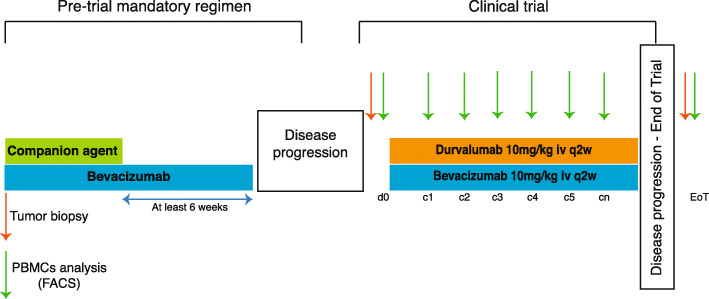
Trial design. Patients had to be receiving maintenance treatment with bevacizumab alone as a part of a previous bevacizumab-containing regimen for advanced disease, after discontinuing the companion agent because of the usual clinical practice reasons (cumulative toxicity or achievement of maximal disease response). Pre-trial bevacizumab maintenance was allowed at 5 mg/kg weekly, 10 mg/kg q2w, or 15 mg/kg q3w. When patients experienced disease progression while on bevacizumab treatment become candidates for the trial. Bevacizumab treatment was never stopped, and the first durvalumab dose was scheduled for infusion on the next planned bevacizumab dose. All patients switched to a 10 mg/kg weekly bevacizumab schedule in case they were receiving it on a different one. A fresh tumor biopsy was obtained within a time-window of 7 days prior to the first durvalumab dose. In addition, a PBMC sample was obtained on day 1 prior to the first durvalumab dose, and repeated periodically until disease progression; an additional sample was harvested at the end-of-treatment visit (28 days after coming-off trial). Treatment continued until disease progression, unacceptable toxicity or investigator decision

The treatment consisted of 10 mg/kg i.v. of durvalumab plus 10 mg/kg i.v. of bevacizumab every 2 weeks. One cycle was defined as 28 days. Hormone-positive patients were not allowed to receive hormonal therapy while on trial. CT (computed tomography) scans were performed every 8 weeks and disease burden was evaluated according to RECIST 1.1 criteria (Response Evaluation Criteria in Solid Tumors). Toxicity was graded according to NCI CTC AE V.4.03 (Common Terminology Criteria for Adverse Events). Figure 1 depicts basic trial design.

A peripheral blood sample was obtained on the day of the first durvalumab dose in order to analyze peripheral-blood mononuclear cells (PBMCs) subpopulations by flow cytometry (FACS). Blood sampling for FACS analysis was repeated on weeks 4, 8, 12, 16, 20, and 24 and at the end-of-treatment visit. Patients underwent a tumor biopsy between 5 and 7 days prior to the first study treatment.

PBMCs immunophenotyping was performed as follows: blood (~ 30 ml) was collected in CPT Cell Preparation Tubes (BD) and PBMC isolated by centrifugation. Immunophenotyping was performed by staining 2 × 10^6^ PBMCs with a combination of antibodies (Table S1) proposed by the Human Immunology Project Consortium to characterize T cell subtypes, regulatory T cells (T_REG_), dendritic cells (DC), monocytes, and natural killer (NK) cells [27]. Stained samples were acquired in a Gallios cytometer (Beckman Coulter); the gating strategy for each panel was performed with the Kaluza software and is shown in Supplementary Figure S1, S2, S3. The ratio between effector and non-effector T cell subtypes was calculated by considering the non-effector group the sum of the percentages of naïve and central memory T cells (T_CM_), whereas the effector group was constituted by the sum of the percentages of terminally differentiated effector (T_eff_) and effector memory T cells (T_EM_).

Regarding immunohistochemistry studies, archival and baseline tumor samples were assessed centrally for PD-L1 expression with the Ventana SP263 assay (Ventana Medical Systems, Inc., Oro Valley, Arizona). Immunohistochemical stainings were performed on 2.5-μm tissue sections, using an automated protocol developed for the Autostainer Link automated slide staining system (DAKO, Agilent). All steps were performed on this staining platform using validated reagents, including deparaffinization, antigen retrieval (cell conditioning), and antibody incubation and detection. The following antibodies were used for IHC: CD8 FLEX (Clone C8/144B, DAKO; #IR623/IS623), CD4 FLEX (Clone 4B12, DAKO; #IR649), and FoxP3 (Clone 236A/E7, Abcam; ab20034). Corresponding tissue sections were acquired and digitalized using the AxioScan.Z1 system (Zeiss). Digitalized images were automatically analyzed with the ZEN 2.3 lite software (Zeiss). The percentage of FoxP3 positivity was considered as ratio of FoxP3-positive cells on total number of cells.

Combined pericyte and endothelial cell staining for assessing vessel architecture normalization was performed with confocal microscopy. For immunofluorescence staining, deparaffinization and antigen retrieval of 2.5-μm tissue sections were performed. Then slides were washed twice with TBS-T (0.1% Tween 20), and then blocked in TBS-T with 2.5% bovine serum albumin for 30 min before the indicated primary antibody was applied. Endothelial cells were detected with a mouse monoclonal antibody against CD31/PECAM-1 (clone JC/70A, NovusBio; #NB600-562) and pericytes were detected with a rabbit monoclonal antibody against NG2 (clone EPR9195, Abcam; #ab139406). Cells were then washed with TBS-T and incubated with Alexa Fluor 488- or 555-conjugated secondary antibody (Molecular Probes). Cell nuclei were stained with DAPI (4’,6-diamidino-2-fenilindol) (Sigma). After washing, coverslips were mounted onto glass microscope slides with ProLong™ Gold antifade reagent (Invitrogen). Images were acquired using Leica-TCS SP5 MP confocal microscope, with a HCX PL APO 63× 1.4 NA oil-immersion objective using LAS AF version 2.6 software.

Finally, gene-expression studies in tumor samples were performed with RNAseq: total RNA from formalin-fixed paraffin-embedded tissue samples was isolated using the Maxwell® RSC RNA FFPE kit (#AS1440, Promega) with the Maxwell® RSC Instrument (Promega) according to the manufacturer’s protocol. Sequencing libraries were prepared with the “QuantSeq 3′ mRNA-Seq Library Prep Kit” (Lexogen, Cat.No. 015). Directional cDNA libraries are initiated by reverse transcription with oligodT priming and eventually sequenced in single-read format in a HiSeq 2500 instrument (Illumina).

Sequencing read alignment and quantification and differential gene expression analysis was performed in the Bluebee Genomics Platform, a cloud-based service provider (www.bluebee.com). Briefly, reads are first trimmed with bbduk from BBTools (Bushnell B., BBMap, https://sourceforge.net/projects/bbmap/) to remove adapter sequences and polyA tails. Trimmed reads are aligned to the GRCh38/hg38 genome assembly with STAR v 2.5. Read counting is performed with HTSeq and differential gene expression analysis, between the responder and non-responder conditions, is done with DESeq2. Gene set enrichment analysis (GSEA) versus the ImmuneSigDB molecular signatures database [28] was performed on a ranked list of DESeq2 data, where log2FC of genes showing greater than 1.2 absolute fold change was divided by their corresponding *p* value.

### Aims and statistical analysis

The sample size was designed to study immunodynamics in peripheral blood and in tumor, while gathering efficacy and toxicity data. Thus, the primary aim was to compare different PBMC subpopulations (at baseline or during treatment) among patients showing benefit or not from the combination. Since the patients enrolled in this trial were at advanced treatment lines and one of the two study drugs was one to which they had already experienced treatment failure (bevacizumab), we considered that those patients that did not experience disease progression at the standard landmark for immune-oncology drugs evaluation time (16 weeks) were experiencing clinical benefit of the study drugs. Those patients were termed “non-progressors,” as opposed to those showing progressive disease at week 16 (“progressors”) for biomarker analysis purposes. Sample size was determined according to the ability to detect at least a 10% difference in any given PBMC subpopulation between progressors and non-progressors. Setting alpha and beta in 5% and 80%, respectively, a minimum of 24 patients was deemed necessary to discriminate such effect. All patients receiving at least one dose of study treatment were included in the safety and efficacy analysis.

The co-primary aim was to determine the overall survival (OS) and progression-free survival (PFS). Secondary aims were to study the safety and toxicity of the combination.

The differences between the percentages of leukocyte subtypes between progressors and non-progressors were compared using a two-tailed Student’s *t* test after Arcsin transformation of the data. Variances were compared with the *F* test, and Welch’s correction applied when suspected to have not-equal standard deviation. CD4, CD8, and T_regs_ infiltration; PD-L1 staining; and vascular normalization were compared with a T-test. OS and PFS estimates were compared with the log-rank test and Kaplan-Meier curves. All tests were two-tailed and performed with SPSS V.19 software.

## Results

### Patients and treatment

**Fig. 2:**
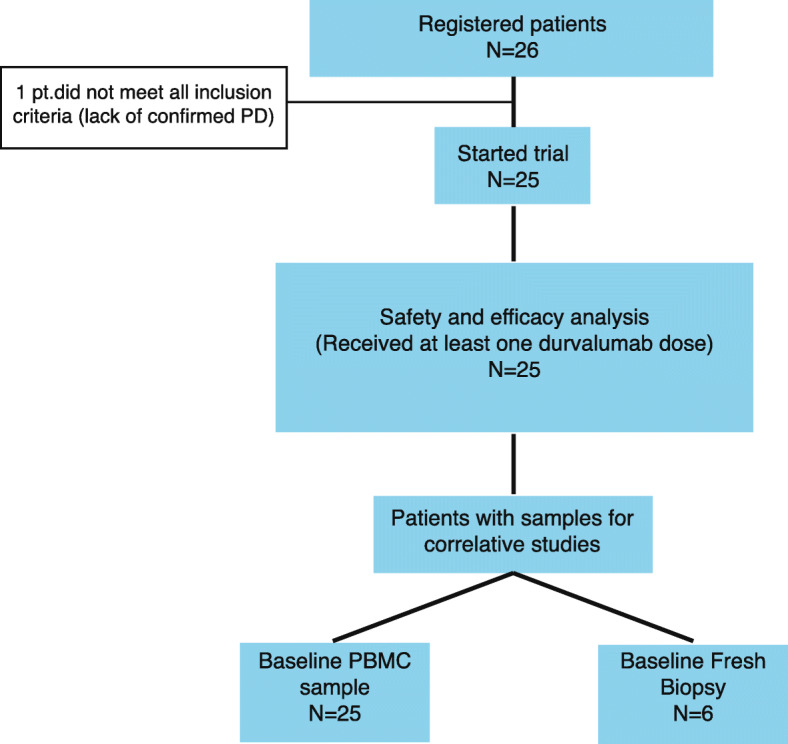
CONSORT diagram. Twenty six patients underwent trial screening but one patient was deemed ineligible since she was not found to have documented progressive disease to ongoing bevacizumab maintenance. The rest of the patients (*N* = 25) received at least 1 durvalumab dose and were included in the safety and efficacy analysis. All patients had at least one baseline PBMCs sample for the immune subpopulation analysis; of them, only 6 consented for the pre-treatment tumor biopsy that was subsequently used for immunohistochemistry and gene-expression studies

From June 2016 to July 2018, 26 patients were accrued at the 8 study sites. Their basic demographic and clinical characteristics are shown in Table 1. One patient was found to not having a documented disease progression to bevacizumab maintenance before entering the trial and was excluded from the analysis. On average, patients had been exposed to 8.4 months of continuous bevacizumab dosing before registration. Patients had been on up to 7 lines of therapy for metastatic disease. Approximately 2/3 and 1/3, respectively, were hormone-positive and triple-negative breast cancer patients (Table 1). A CONSORT diagram describing trial accrual and populations for safety, efficacy, and correlative studies is shown in Fig. 2.

**Table 1:** Demographic and baseline clinical characteristics

Characteristic	Value
Age (median, range)	54.1 (34.5–77.4)
Tumor subtype	
Hormone-positive	16 (64%)
TNBC	9 (36%)
Histology	
Ductal	18 (72%)
Lobular	4 (16%)
Other	3 (12%)
Time (months) from diagnosis of metastatic disease (median, range)	18.1 (2–116)
Time (months) receiving bevacizumab before registration (median, range)	8.4 (1.6–24.7)
Number of previous treatment lines for metastatic disease	
1	3 (12%)
2	9 (36%)
3	3 (12%)
4	7 (28%)
5	2 (8%)
7	1 (4%)
Companion drug in the pre-trial regimen:	
Capecitabine	13 (52%)
Paclitaxel	10 (40%)
Docetaxel	1 (4%)
Cisplatin	1 (4%)
ECOG 0	15 (60%
ECOG 1	10 (40%)
PD-L1 expression:	
Positive (> 1%)	4 (16%)
Negative (0%)	16 (64%)
Unknown	5 (20%)

### Efficacy

**Fig. 3:**
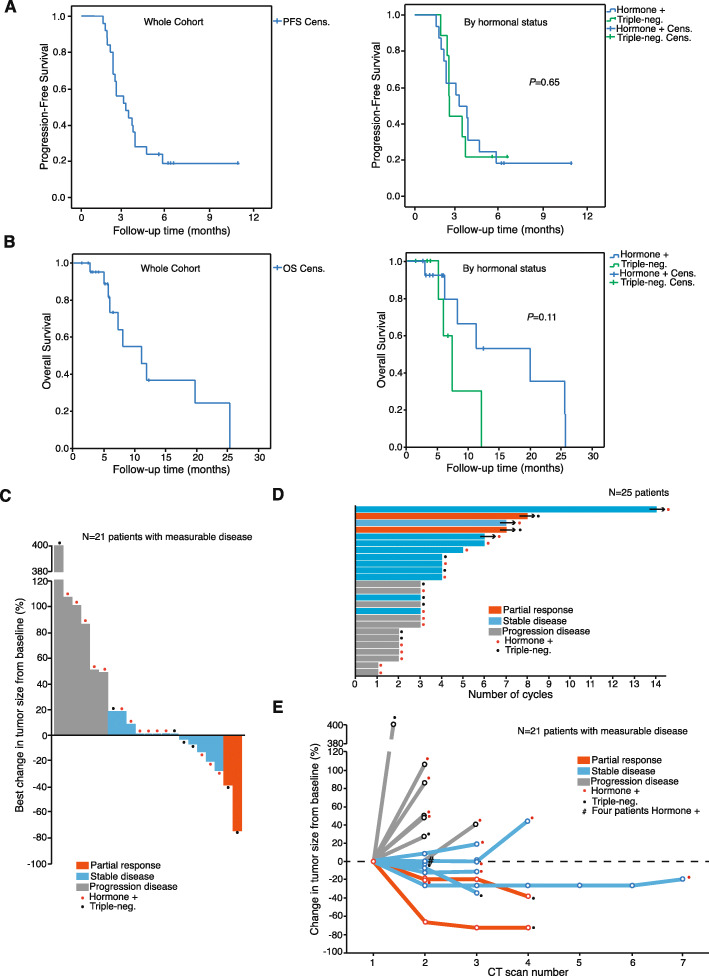
Overall efficacy data. **a** Kaplan-Meier curves (PFS) for the whole population (left) and split by hormonal subtype (TNBC, 2.6 months; hormone-positive, 3.3 months; log-rank *P* value, 0.84). **b** Kaplan-Meier curves (OS) for the whole population (left) and split by subtype (TNBC, 7.4 months; hormone-positive, 19.8 months; log-rank *P* value, 0.11). **c** Waterfall plot showing best percentage change from baseline in the sum of the longest diameters of target lesions. This plot depicts the changes among the *N* = 21 patients with measurable disease; the remaining patients (*N* = 4) had bone disease only (non-measurable but evaluable disease, according to RECIST 1.1). The patient that was non-evaluated due to clinical PD was one of the patients with non-measurable disease. **d** Swimmer plot depicting the time and durability of response of the 25 patients included in the trial. **e** Spider plot displaying the longitudinal change from baseline in the sum of the longest diameters of target lesions. It depicts the changes among the same *N* = 21 patients as in (**c**). Patients in (**c**), (**d**), and (**e**) are labeled according to their hormonal status: red dot, hormone-positive; black dot, TNBC

Database lock was performed on February 2019, when patients had been receiving the study regimen for a median of 13 weeks (range 4.1 to 57.3 weeks). At this timepoint, 5 patients remained on active treatment for 24+, 28+, 28+, 32+, and 58+ weeks. Median progression-free survival (PFS) and overall survival (OS) were 3.5 months and 11 months, respectively (Fig. 3a and b). PFS was not significantly different for TNBC (triple-negative breast cancer) and hormone-positive patients (Fig. 3a). Regarding OS, there was a trend for improved outcomes for the hormone-positive patients (median OS for TNBC, 7.4 months; median OS for hormone-positives, 19.8 months; *P* = 0.11; Fig. 3b). The best changes in disease burden and disease burden changes over time among the 21 patients with measurable disease are shown in the waterfall plot and spider plot (Fig. 3c and e). Overall, 28% of the patients (*N* = 7) experienced some degree of tumor shrinkage (although 5 of the 7 patients with tumor shrinkage did not achieve partial response criteria according to RECIST 1.1; Fig. 3c). The changes in tumor burden were sustained for at least two cycles among most of the patients achieving SD or PR, as shown in Fig. 3d. Clinical benefit (CR+PR+SD) was observed in 15/25 patients (clinical benefit rate − CBR − 60%) at 8 weeks; at 16 weeks, the CBR was 44%. Patients experiencing SD or PR showed similar disease-control duration (Fig. 3e). No complete responses were observed. Best objective overall responses were as follows: PR was observed in 2 patients (8%); SD was registered in 10 (40%); with the exception of one patient that did not reach the first CT scan (4%, non-evaluated; clinical PD), the remaining 12 patients (48%) experienced PD (total number of PD: *N* = 13; 52%). According to the hormonal status, hormone-positive patients achieved SD in 6/16 cases (37.5%), whereas the rest of the patients (10/16; 62.5% patients) showed PD as best response (one of them was not evaluated but experienced clinical PD). The two observed responses (2/9, 22.2%) occurred in TNBC patients; 2 additional TNBC patients (22.2%) experienced SD, whereas the remaining TNBC patients (*N* = 5; 55.6%) experienced PD as best response. The comparison between TNBC and hormonal patients regarding the CBR did not show statistically significant differences (*P* = 0.73).

### Differences in peripheral blood mononuclear cells (PBMCs) immunophenotyping between progressors and non-progressors

**Fig. 4:**
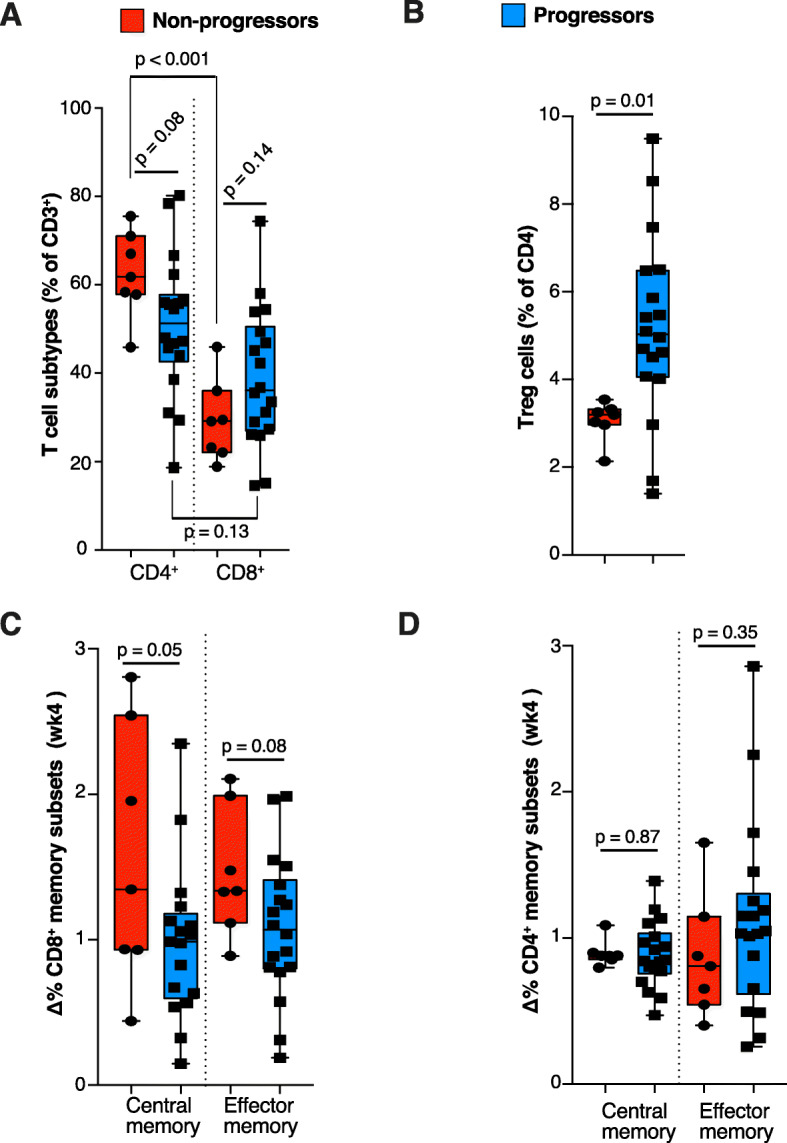
Immune cell sub-populations associated to clinical benefit in peripheral blood mononuclear cells. **a** Frequency of T-cell subtypes determined by immunophenotyping of PBMC from peripheral blood in non-progressors (red) and progressors (blue) patients in the baseline sample. **b** Differential T_reg_ percentage in baseline samples among progressors and non-progressors. **c** Non-progressors displayed a significant increase in central memory—and a trend to increase in effector memory—CD8^+^ T-cells 4 weeks after the first treatment dose, compared with progressors. **d** These changes were not evident for CD4^+^ T cells

Seven patients were classified as “non-progressors” whereas eighteen were classified as “progressors” (statistics section). The PBMCs analysis of the baseline samples (before the first durvalumab dose; *N* = 25) yielded the following results: first, no differences in the major generic populations (T cells, monocytes, DCs and NK cells) among progressors and non-progressors (Figure S4A) were found. In addition, no baseline differences were evident either in innate immune populations (Figure S4B, C). Conversely, detailed analysis of T cell subtypes showed an unusually high frequency of CD4^+^ T cells in the blood of non-progressors compared to that of progressors (Fig. 4a). Interestingly, we observed a strong reduction in the baseline percentage of circulating immunosuppressive T regulatory (T_reg_) cells in non-progressors compared to progressors (Fig. 4b). No other differences between progressors and non-progressors were evident for naïve, effector (T_eff_), effector memory (T_EM_) and central memory (T_CM_) CD4^+^ or CD8^+^ T cell subtypes (Figure S4D, E). The ratio of effector (T_eff_ + T_EM_) and non-effector (T_naïve_ + T_CM_) populations were not correlated with the clinical outcome (Figure S4F, G) either.

Regarding the longitudinal analyses in on-treatment samples, it was revealed that CD8^+^ T_EM_ and CD8^+^ T_CM_ were increased after the first durvalumab dose compared to the baseline levels (Fig. 4c); this pattern was attenuated along subsequent cycles and not observed for CD4^+^ memory subsets (Fig. 4d). No other clear differences between non-progressors and progressors in the frequency of specific T-cell or innate subpopulations (Figure S5) were found.

### Gene-expression patterns and vascular normalization in tumors from progressors and non-progressors

**Fig. 5:**
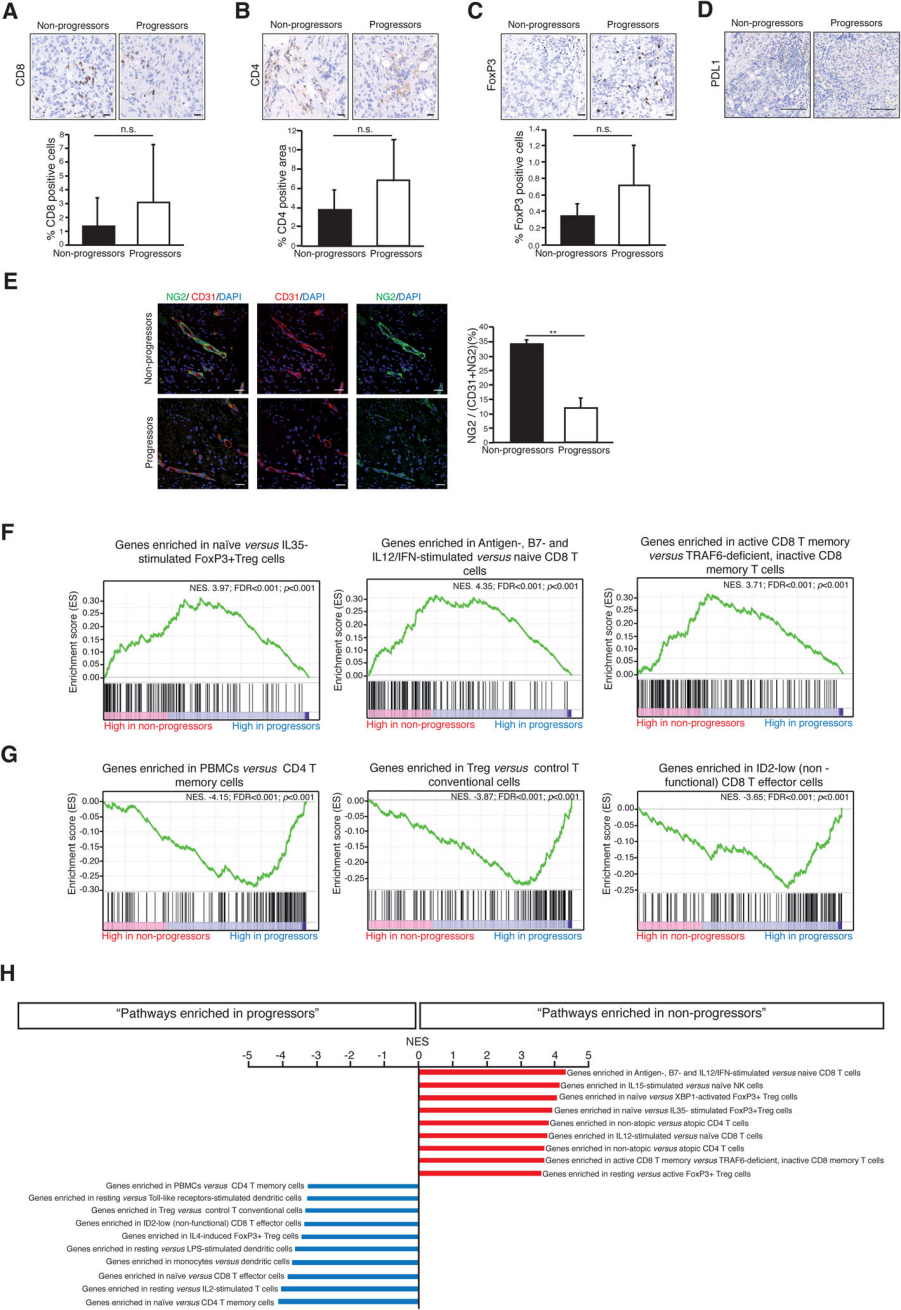
Tumor tissue immunodynamics and gene expression studies suggest immuno-priming by bevacizumab. Representative immunohistochemistry images of **a** CD8^+^ infiltration, **b** CD4^+^ infiltration, and **c** T_reg_ infiltration in tumors from non-progressor and progressors patients (upper panels). The lower panels represent the geometric mean and standard deviation of the quantitation data from all available tumor biopsies. **d** The six analyzed tumors were negative for PD-L1 expression (applying the 1% boundary), regardless of experiencing benefit or not from the treatment combination. **e** Confocal imaging showing representative fields containing normalized blood vessels from a non-progression (the whole microvessel wall—CD31-positive endothelial cells—is covered by NG2-positive pericytes) and a progressor patient (who, in turn, displays vessel abnormality—lack of pericyte coverage and tortuous architecture). The chart represents the quantitative differences between the average percentage of microvessel wall covered by pericytes in non-progressors versus progressors; **P < 0.01. **f** Functionally representative GSEAs of the main regulated pathways in non-progressors’ tumors; NES, normalized enrichment score (the higher NES, the higher functional enrichment); both corrected (false discovery ratio (FDR)) and uncorrected *P* values are shown. **g** Same as in (**f**) for non-responders. **h** Further enriched GSEAs in responders and non-responders ranked by their NES; all of them with FDR < 0.001. Scalebars: **a**–**c** 20 μm; **d** 100 μm; **e** 25 μm

In an attempt to understand the PBMCs findings in the context of the mechanism of action of the combination, we studied tumor biopsies. Six patients (3 non-progressors and 3 progressors) had available a baseline tumor biopsy (i.e., after bevacizumab exposure and before the first durvalumab dose). Non-progressors’ tumors did not show significant differences in CD8, CD4, or T_reg_ infiltration compared to the progressors (Fig. 5a–c). In addition, the six baseline biopsies were negative for PD-L1 staining (Fig. 5d). Morphologically, however, the examination of the vascular network of the baseline samples displayed pericyte coverage of the endothelial cells in non-progressors as opposed to progressors (Fig. 5e), indicating vascular normalization in response to pre-trial bevacizumab exposure in the former [29].

Despite the lack of meaningful morphological or numerical differences in the tumor immune infiltrate, gene expression revealed profound differences in its functional status. When gene expression levels were queried for gene set enrichment (GSEAs) against the ImmuneSig database [28], we found the following differences in immunological pathways between non-progressors and progressors: First, congruently with the observations in peripheral blood, non-progressors displayed a reduction of GSEAs related with suppressive activity of FoxP3 T_reg_ cells, but an enrichment in signatures related to stimulated CD8 T cells (Fig. 5f and h), which suggest an increased effector activity in the tumor tissue. Conversely, enrichment in active FoxP3 T_reg_ and inactive CD8 cells signatures were observed in progressors (Fig. 5g and h). In addition, we observed an enrichment in GSEAs of unstimulated resting DCs in progressors versus non-progressors’ tumors (Fig. 5h). Finally, memory T cell GSEAs were increased in non-progressors versus progressors patients (Fig. 5f and h).

### Safety

One hundred and six cycles have been administered. Table 2 shows the incidence of grade 1–2 adverse events related with the study drugs. There were two grade 3 events possibly related to the study drugs, registered in the same patient: a patient with lung lymphangitic carcinomatosis and previous history of asthma and smoking habit was admitted with grade 3 respiratory insufficiency, grade 3 bronchospasm, and grade 2 infection during cycle 2. She received supportive measures, antibiotics, and prednisone and continued on durvalumab + bevacizumab after discharge without further side effects. The patient was diagnosed as COPD exacerbation; thus, the relationship with the study drugs remains unclear. No grade 4 events were registered. Four events qualified as severe adverse events (SAEs): three of them each registered and deemed related to disease progression in three patients (pain worsening, pancreatitis in a patient with a pancreatic metastasis, and general status decline); the last SAE corresponded to the patient with grade 3 side effects.

**Table 2:** Adverse events (grades 1 and 2)

Event	Grade 1	Grade 2	Total
Asthenia	4 (16%)	1 (4%)	5 (20%)
Headache	2 (8%)	1 (4%)	3 (12%)
Proteinuria	2 (8%)	0 (0%)	2 (8%)
Hypertension	1 (4%)	1 (4%)	2 (8%)
Diarrhea	2 (8%)	0 (0%)	2 (8%)
Pruritus	0 (0%)	1 (4%)	1 (4%)
Hyporexia	0 (0%)	1 (4%)	1 (4%)
Nausea	0 (0%)	1 (4%)	1 (4%)
Pneumonitis	0 (0%)	1 (4%)	1 (4%)
Thrombosis	0 (0%)	1 (4%)	1 (4%)
Infection	0 (0%)	1 (4%)	1 (4%)
Elevated amylase	1 (4%)	0 (0%)	1 (4%)
Elevated lipase	1 (4%)	0 (0%)	1 (4%)
Rash	1 (4%)	0 (0%)	1 (4%)
Xerosis	1 (4%)	0 (0%)	1 (4%)
Hypothyroidism	1 (4%)	0 (0%)	1 (4%)
Abdominal pain	1 (4%)	0 (0%)	1 (4%)
Vomiting	1 (4%)	0 (0%)	1 (4%)
Pyrexia	1 (4%)	0 (0%)	1 (4%)
Arthralgia	1 (4%)	0 (0%)	1 (4%)
Xerophtalmia	1 (4%)	0 (0%)	1 (4%)

## Discussion

In order to ascertain the potential priming role of antiangiogenic treatment for subsequent immunotherapy, we conducted a pilot biomarker and efficacy study in advanced HER2-negative breast cancer patients. Preclinical research suggests that antiangiogenic agents could exert a priming of the immune microenvironment [12–15, 17–19, 30]. Particularly, the sequence in which these agents are administered (sequential versus concurrently with immunotherapy) might be of key importance [25, 26]. The efficacy assessment and the correlative studies results deserve attention.

In our heavily pretreated population, we observed a PFS of 3.5 months and an OS of 11 months. The response rate according to RECIST 1.1 was low (8%), but the combination achieved a CBR of 60% at 8 weeks and 44% at 16 weeks. The CBR was not significantly different among TNBC- and hormone-positive patients. Current evidence suggests that immune checkpoint inhibitors are more effective in TNBC [31, 32]. We only observed tumor responses in TNBC; conversely, OS was longer in the hormonal subtypes. Our sample size precludes definitive conclusions about the long-term efficacy of the combination, particularly regarding a selective effect of the combination over one subtype or the other. Most likely, such overall survival differences may be explained by the intrinsic biologic differences between TNBC and the hormonal subtype, which is more benign and usually displays a 2- to 3-fold longer overall survival in the metastatic setting. Regardless, the CBR compares favorably in relation with the existing evidence with PD-1/L1 inhibitors monotherapy in similar populations. Although trial-to-trial comparisons have to be performed with extreme caution, the results of PD-1/L1 inhibitors monotherapy trials are as follows: in a single-agent pembrolizumab study in PD-L1, hormone-positive breast cancer showed a 20% 16-weeks CBR [4]. Two trials that accrued a mixed population of hormone-positive and TNBC patients, testing the efficacy of durvalumab plus tremelimumab [5] and avelumab [3] respectively, achieved a < 30% 4-month CBR. In a monotherapy trial with pembrolizumab in previously untreated TNBC, the disease control rate was 23.8% [1], whereas in a previously treated cohort this rate decreased to just 7.6% [2]. The 4-month CRB observed in our heavily pretreated cohort is encouraging and could be supporting the immuno-priming effects of bevacizumab. However, our design does not distinguish between “priming” effects (versus no-priming) or even whether it is justified or not to maintain bevacizumab in the combination phase. A definitive conclusion in this regard would require a 2 × 2 randomized design comparing the inclusion or not of a bevacizumab priming phase followed by durvalumab monotherapy or in combination with bevacizumab, together with pre-priming, pre-durvalumab, and on-treatment PBMC serial sampling. A recent randomized trial in advanced HER2-negative disease without targetable molecular alterations in patients not showing progressive disease after 6–8 chemotherapy cycles found a lower progression-free survival but longer overall survival of durvalumab compared to maintenance chemotherapy apparently due to the TNBC patients that were PD-L1 positive [33]. Thus, the role of durvalumab is still unclear in advanced HER2-negative breast cancer, and a randomized study should have a sufficiently large size in order to be able to stratify patients according to their hormonal status and PD-L1 expression. Given the current indications of bevacizumab in breast cancer, and the fact that the present study was an investigator-initiated trial with limited funding, our study had to rely necessarily on patients currently receiving maintenance bevacizumab and seek for a signal of biologic plausibility for the priming effects in the set of correlative studies. That restrictive inclusion criteria may constitute on itself a selection bias towards patients with relatively indolent disease, who do not show immediate progression while in maintenance bevacizumab (evidenced by the average time of 8.4 months before entering the trial under bevacizumab-based regimens; Table 1). In fact, bevacizumab itself might not be the most effective VEGF-targeting drug [21–24] at least in breast cancer, but the investigator-initiated nature of this trial made bevacizumab the only realistic antiangiogenic option for the study. The impressive effects of other antiangiogenics with high affinity against VEGFR2 such as lenvatinib (in gastric [34] and endometrial cancer [35]) or axitinib (in kidney cancer [36]) in combination with pembrolizumab should make at least consider these partners in for combination with immunotherapy in future trials in breast cancer exploring this concept.

The immune-priming hypothesis to explain the clinical benefit from the combination is further supported by the immunologic biomarkers found in peripheral blood. Although current evidence of a response-prone immune-environment (in PBMCs) before treatment with PD-1/L1 inhibitors is lacking in breast cancer [1–5], studies in peripheral blood in other tumor types have shown biomarkers of benefit from immunotherapy [37–39]. Our patient population had been exposed to up to 25 months to bevacizumab, and at this point, patients that experienced benefit from subsequent durvalumab displayed an obvious phenotype consisting in a decreased amount of immunosuppressive T_reg_ cells in peripheral blood (Fig. 4). Those patients that had available tissue biopsy showed a highly congruent gene-expression program, consisting on decreased expression of gene sets related to T_reg_ cells in the tumor microenvironment (Fig. 5f–h). Different studies have established a correlation between aberrant VEGF expression and the proliferation of Treg cells in the tumor microenvironment [40–42]. Our results not only support this local intratumor effect of VEGF blockade on T_reg_ expansion, but suggest that this effect might be systemic given the low frequency of T_reg_ cells in peripheral blood of non-progressors as well. A future randomized study should confirm whether or not the low number of Treg cells in these patients is a direct consequence of previous bevacizumab therapy.

Vascular normalization is the expected positive effect that translates pharmacodynamic engagement by antiangiogenics [16]. The fact that the patients that experienced benefit from the combination and showed the described T cell phenotype in tumor and peripheral blood were also those that experienced a vascular normalizing response to previous bevacizumab exposure (Fig. 5e) further supports the hypothesis that antiangiogenics can exert an immuno-priming effect, at least in some cases. Obviously, not all patients experience vascular normalization in response to antiangiogenics, which seems to be a factor required for immuno-priming [17, 18, 30]. We have previously shown that the normalization rate is agent-dependent (ranging from 1/3 of the patients with bevacizumab [43] to up to ¾ of the patients with nintedanib [44], in HER2-negative breast cancer). These differences should be taken into account for designing future trials. In any case, future biomarker-based studies in this context should be guided by peripheral blood sampling; access to fresh tumor biopsies in metastatic patients is usually limited (only 25% of our patients consented to fresh biopsy), and thus, conclusions may not be extrapolable to real-world patients.

Finally, the observed GSEAs suggesting a dysfunctional DC phenotype in tumors from progressors (Fig. 5h) are congruent with the role of VEGF in DCs’ maturation (progressors also did show vascular abnormality, translating inadequate effect in clearing VEGF by bevacizumab, which alters DC maturation) and the lack of benefit from durvalumab. Tolerogenic DCs are associated to the expansion and differentiation of Treg cells [45]. The observed decrease of T_reg_ signatures in the tumor microenvironment might thus be associated to improved DC maturation.

The main weaknesses for interpretation of our data are patient heterogeneity and the limited sample size. The heterogeneity was not only originated by mixing TNBC and hormone-positive patients: trial patients received a variable number of treatment lines prior to maintenance with bevacizumab and also were on maintenance for highly variable times (from 1.6 to 24.7 months). Whereas patients with many previous treatment lines and long bevacizumab maintenance time might be inherently more indolent than the remaining, disease progression also selects for cancer features associated with refractoriness to immunotherapy [46–48]. Trials evaluating PD-1/L1 inhibitors in more advanced disease lines have shown limited activity compared with earlier lines [1, 2, 31]. Taken together, these features might complicate the interpretation of the overall efficacy data and the immunology correlative studies, at the PBMCs and tumor infiltration levels.

Importantly, no new safety concerns related to durvalumab were detected (Table 2). Although the sample size is relatively low, all the observed toxic events fit into those expected either for bevacizumab or durvalumab and do not suggest any negative drug-drug toxic interaction. It has been proposed that antiangiogenics can decrease the amount and severity of immune-related adverse events (IRAEs) [12]. This trial was not designed for prospective identification of IRAEs; however, the 8% incidence of diarrhea and 4% incidence of rash (plus 2 grade 3 respiratory events in the same patient, with unclear relationship to study drugs)—the side effects traditionally most frequently classified as IRAEs—is not particularly high.

Several trials have studied the simultaneous administration of antiangiogenics plus immune checkpoint inhibitors in different malignancies [36, 49, 50]. To our knowledge, we are the first in reporting on the sequential combination in breast cancer. Our immunophenotyping analyses highlight the predictive role of specific T cell subtypes for the clinical response to PD-L1 blockade, pointing towards several effector T-lymphocyte subpopulations as biomarkers of activity (Fig. 4). In the past, single-agent immunotherapy studies in breast cancer have not shown clear PBMC subpopulations predicting benefit, suggesting that the response-prone environment is not obvious in peripheral blood. The fact that in our study non-progressors showed a differential PBMC pattern compared to the remaining patients after previous exposure to bevacizumab, together with the concurrence of vascular normalization and gene expression programs associated to activated T_eff_ cells and reduced immunosuppressive T_reg_ in their tumors, suggests a positive immune-priming effect of bevacizumab. Taken together the positive efficacy signal, the low toxicity rates, the finding of candidate biomarkers of activity in peripheral blood, and the biological rationale, our data justify a larger prospective clinical trial aiming to define the magnitude of the benefit derived from antiangiogenic priming in this setting.

## Conclusions

The combination of bevacizumab and durvalumab shows promising efficacy in a heavily pre-treated cohort of HER2-negative breast cancer patients previously exposed to chronic bevacizumab. Peripheral blood and tumor immunodynamics suggest that pre-treatment with bevacizumab immuno-primes at least a fraction of patients, making them more prone to benefit from durvalumab. A prospective randomized trial should confirm the role of T_regs_ as potential biomarkers of activity and the magnitude of benefit of this chemo-free regimen.

## Supplementary information

**Additional file 1 : Supplementary Figure S1**: Flow cytometry gating strategy for T cells. Representative gating strategy used in T cell immunophenotyping based on the exclusion of dead cells, the selection of the lymphoid cells by size and complexity, and then the surface expression of CD3, CD4, CD8, CCR7 and CD45RA. The combination of these markers allowed the identification of naïve cells (CCR7^+^CD45RA^+^), T_eff_ (CCR7^−^,CD45RA^+^), T_CM_ (CCR7^+^CD45RA^−^), and T_EM_ (CCR7^−^CD45RA^−^). Staining with the CD38 marker allowed the identification of activated cells, which were minimally detected only in T_eff_ and T_EM_ subpopulations.

**Additional file 2 : Supplementary Figure S2:** Flow cytometry gating strategy for Treg cells. Representative gating strategy used in T_reg_ cell immunophenotyping based on the exclusion of dead cells, the selection of the lymphoid cells by size and complexity, and then the surface expression of CD3, CD4, CD25, CD127, CCR4, HLA-DR and CD45RO markers. After the initial selection of CD4^+^ T cells (CD3^+^CD4^+^), T_reg_ cells were identified as CD25^+^CD127^low^ double positive cells. The combination of CCR4 and CD45RO markers allowed the identification of naïve T_reg_ (CCR4^+^,CD45RO^−^) and memory T_reg_ (CCR4^+^CD45RO^+^), whereas HLA-DR positivity identified activated cells in these T_reg_ subpopulations.

**Additional file 3 : Supplementary Figure S3**: Flow cytometry gating strategy for innate cell populations. Representative example of the gating strategy used for immunophenotyping of the indicated innate subpopulations. After the exclusion of dead cells, monocytes were selected by size and complexity; subtypes were identified by CD14 and CD16 staining, allowing the identification of classical monocytes (CD14^+^ CD16^−^), alternative monocytes (CD14^−^CD16^+^) and intermediate monocytes. For DC and NK, leukocytes were selected by size and complexity in the live cells, and T- and B-lymphocytes excluded by staining with lineage-specific antibodies. Dendritic cells (DC) and NK cells were selected within the CD20^−^ and CD14^−^ population; NK cells were identified as CD56^+^ cells (both CD16^+^ and CD16^−^) and DC as HLA-DR^+^CD16^−^ cells. DC subtypes were further defined by CD11c^+^ (myeloid DC) or CD123^+^ (plasmacytoid DC).

**Additional file 4 : Supplementary Figure 4**: Immunophenotyping of leukocyte populations in the baseline sample of responders and non-responder patients. **(A-D)** Analysis of the indicated leukocyte subtypes in the baseline blood sample of the patients stratified according to their clinical response. The percentages of the main leukocyte subtypes (*A*), CD4^+^ and CD8^+^ T cells (*B*), monocyte subtypes (*C*) and DC subtypes (*D*) are shown.

**Additional file 5 : Supplementary Figure 5:** Longitudinal effects of treatment on leukocyte populations**.** For each subpopulation, the time-dependent increment or decrement during treatment is shown, using the baseline sample as reference. Red charts: responders; blue charts: non-responders.

**Additional file 6 : Supplementary Table 1:** Antibodies used for immunophenotyping in peripheral blood.

## Data Availability

The results from gene-expression studies by RNA-seq in tumor samples were deposited in Gene Expression Omnibus (GEO, NCBI) under the accession number GSE139050 .

## Abbreviations

PBMCs: Peripheral-blood mononuclear cells
OS: Overall survival
PFS: Progression-free survival
TMB: Tumor mutational burden
VEGF: Vascular Endothelial Growth Factor
DC: Dendritic cell
ECOG: Eastern Cooperative Oncology Group
LVEF: Left ventricular ejection fraction
CT scan: Computed tomography scan
RECIST 1.1: Response Evaluation Criteria in Solid Tumors
NCI CTC AE V.4.03: Common Terminology Criteria for Adverse Events
FACS: Flow cytometry
CPT: Cell Preparation Tubes
T_REG_: Regulatory T cells
NK: Natural killer cells
T_CM_: Central memory T cells
T_eff_: Terminally differentiated effector T cells
T_EM_: Effector memory T cells
IHC: Immunohistochemistry
DAPI: 4’,6-Diamidino-2-fenilindol
GSEA: Gene set enrichment analysis
TNBC: Triple-negative breast cancer
CBR: Clinical benefit rate
CR: Complete response by RECIST 1.1
PR: Partial response by RECIST 1.1
SD: Stable disease by RECIST 1.1
PD: Progressive disease by RECIST 1.1
COPD exacerbation: Chronic obstructive pulmonary disease exacerbation
SAEs: Severe adverse events
IRAEs: Immune-related adverse events
