# Supplementary figures and images for: Immuno-priming durvalumab with bevacizumab in HER2-negative advanced breast cancer: a pilot clinical trial
Source: Breast Cancer Res. 2020 Nov 11;22:124. doi: 10.1186/s13058-020-01362-y (PMC7661209; doi:10.1186/s13058-020-01362-y)

**Figure Supplementary 1**

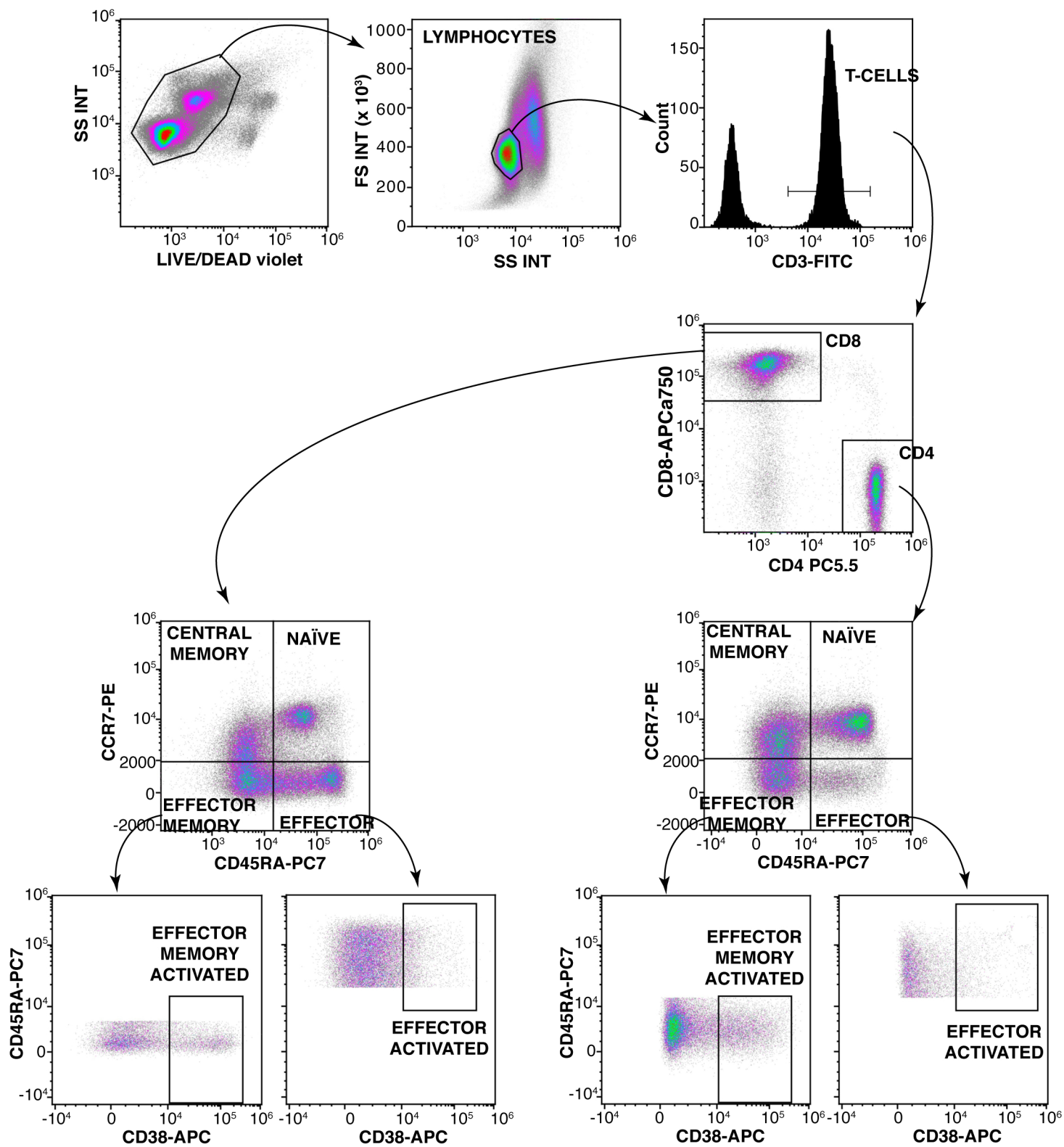

Supplement: Supplementary file 1 — Additional file 1 : Supplementary Figure S1: Flow cytometry gating strategy for T cells. Representative gating strategy used in T cell immunophenotyping based on the exclusion of dead cells, the selection of the lymphoid cells by size and complexity, and then the surface expression of CD3, CD4, CD8, CCR7 and CD45RA. The combination of these markers allowed the identification of naïve cells (CCR7+CD45RA+), Teff (CCR7−,CD45RA+), TCM (CCR7+CD45RA−), and TEM (CCR7−CD45RA−). Staining with the CD38 marker allowed the identification of activated cells, which were minimally detected only in Teff and TEM subpopulations. [file 13058_2020_1362_MOESM1_ESM.pdf]

Figure Supplementary 2

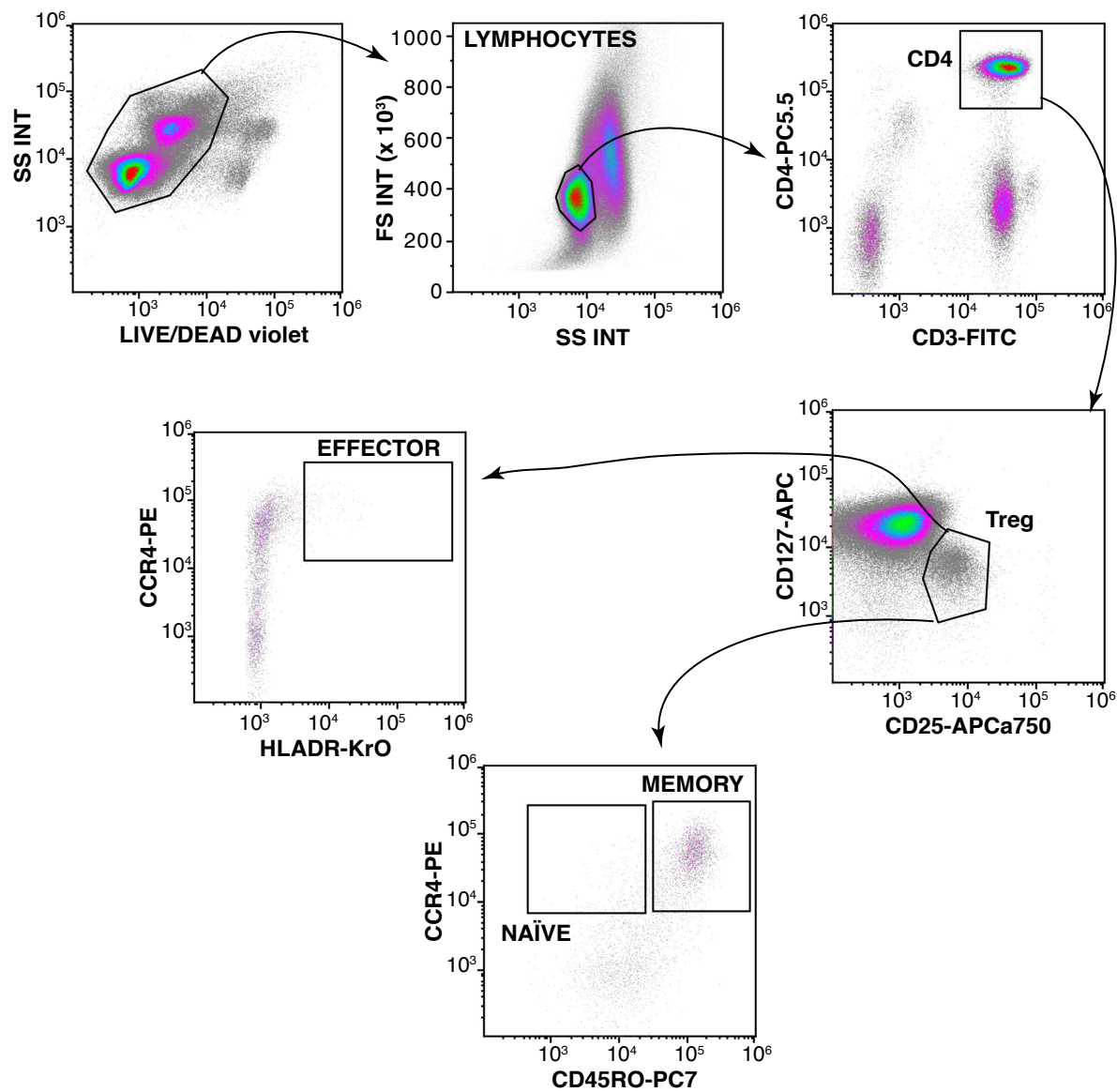

Supplement: Supplementary file 2 — Additional file 2 : Supplementary Figure S2: Flow cytometry gating strategy for Treg cells. Representative gating strategy used in Treg cell immunophenotyping based on the exclusion of dead cells, the selection of the lymphoid cells by size and complexity, and then the surface expression of CD3, CD4, CD25, CD127, CCR4, HLA-DR and CD45RO markers. After the initial selection of CD4+ T cells (CD3+CD4+), Treg cells were identified as CD25+CD127low double positive cells. The combination of CCR4 and CD45RO markers allowed the identification of naïve Treg (CCR4+,CD45RO−) and memory Treg (CCR4+CD45RO+), whereas HLA-DR positivity identified activated cells in these Treg subpopulations. [file 13058_2020_1362_MOESM2_ESM.pdf]

**Figure Supplementary 3**

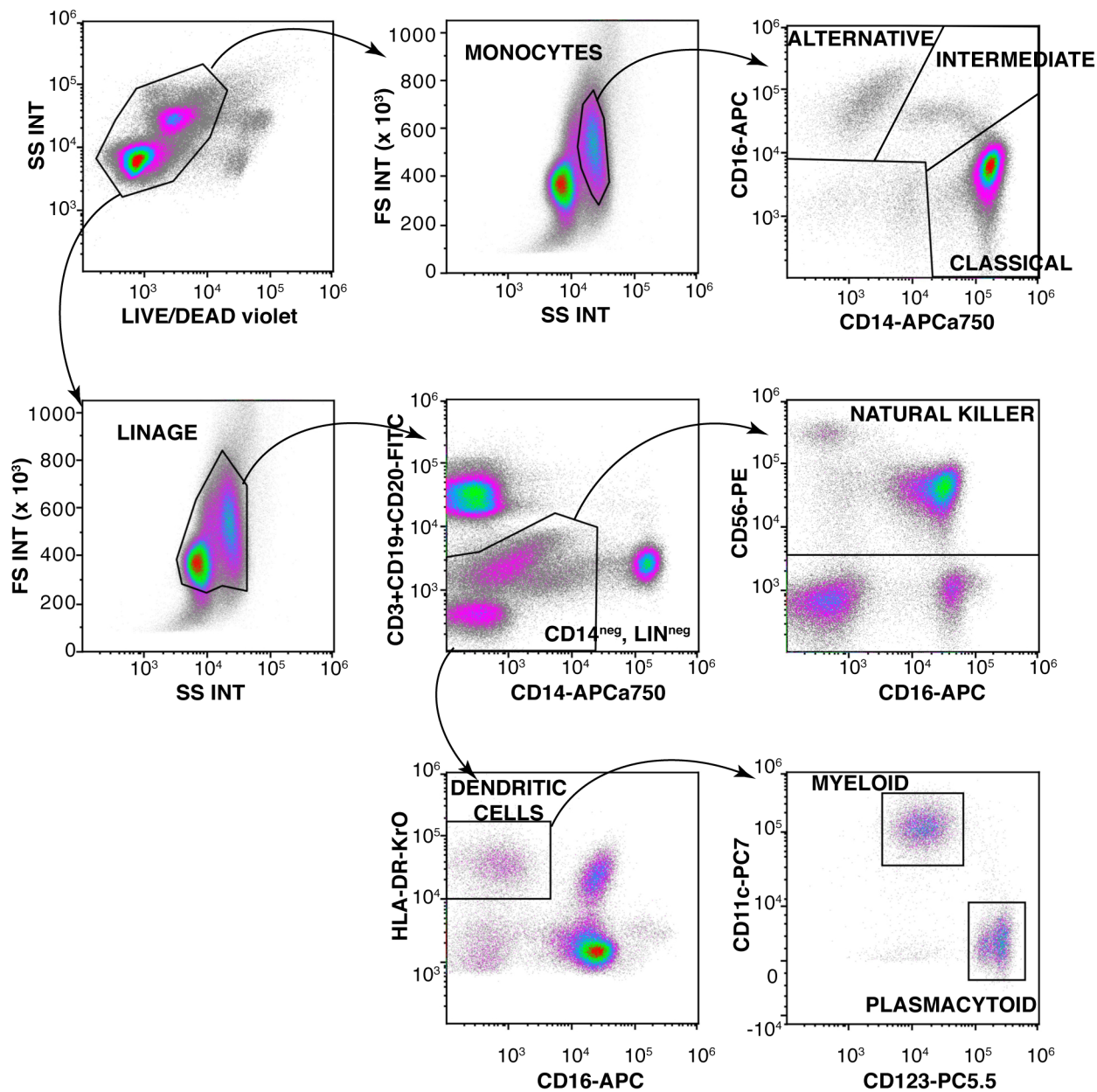

Supplement: Supplementary file 3 — Additional file 3 : Supplementary Figure S3: Flow cytometry gating strategy for innate cell populations. Representative example of the gating strategy used for immunophenotyping of the indicated innate subpopulations. After the exclusion of dead cells, monocytes were selected by size and complexity; subtypes were identified by CD14 and CD16 staining, allowing the identification of classical monocytes (CD14+ CD16−), alternative monocytes (CD14−CD16+) and intermediate monocytes. For DC and NK, leukocytes were selected by size and complexity in the live cells, and T- and B-lymphocytes excluded by staining with lineage-specific antibodies. Dendritic cells (DC) and NK cells were selected within the CD20− and CD14− population; NK cells were identified as CD56+ cells (both CD16+ and CD16−) and DC as HLA-DR+CD16− cells. DC subtypes were further defined by CD11c+ (myeloid DC) or CD123+ (plasmacytoid DC). [file 13058_2020_1362_MOESM3_ESM.pdf]

Figure Supplementary 4

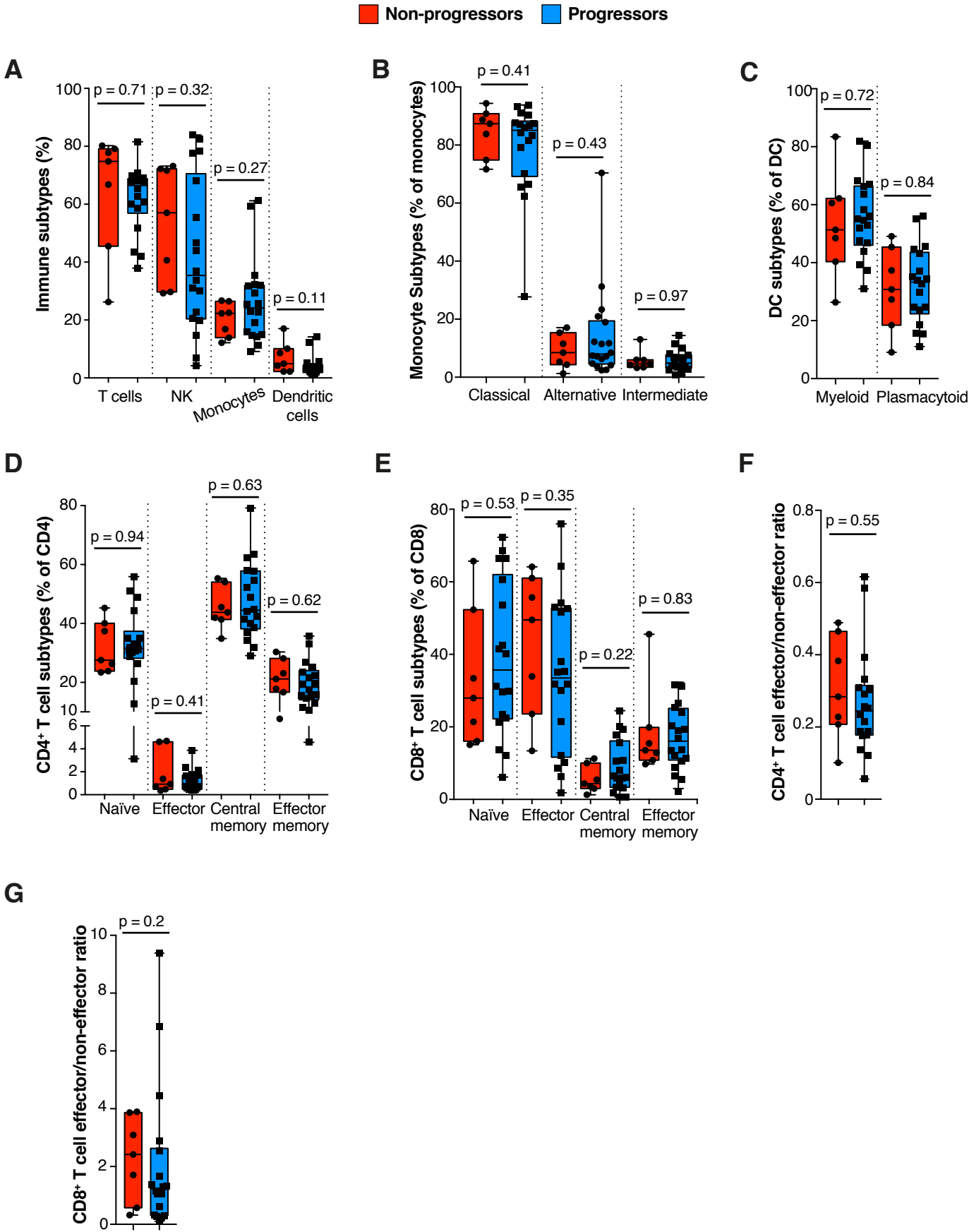

Supplement: Supplementary file 4 — Additional file 4 : Supplementary Figure 4: Immunophenotyping of leukocyte populations in the baseline sample of responders and non-responder patients. (A-D) Analysis of the indicated leukocyte subtypes in the baseline blood sample of the patients stratified according to their clinical response. The percentages of the main leukocyte subtypes (A), CD4+ and CD8+ T cells (B), monocyte subtypes (C) and DC subtypes (D) are shown. [file 13058_2020_1362_MOESM4_ESM.pdf]

Figure Supplementary 5

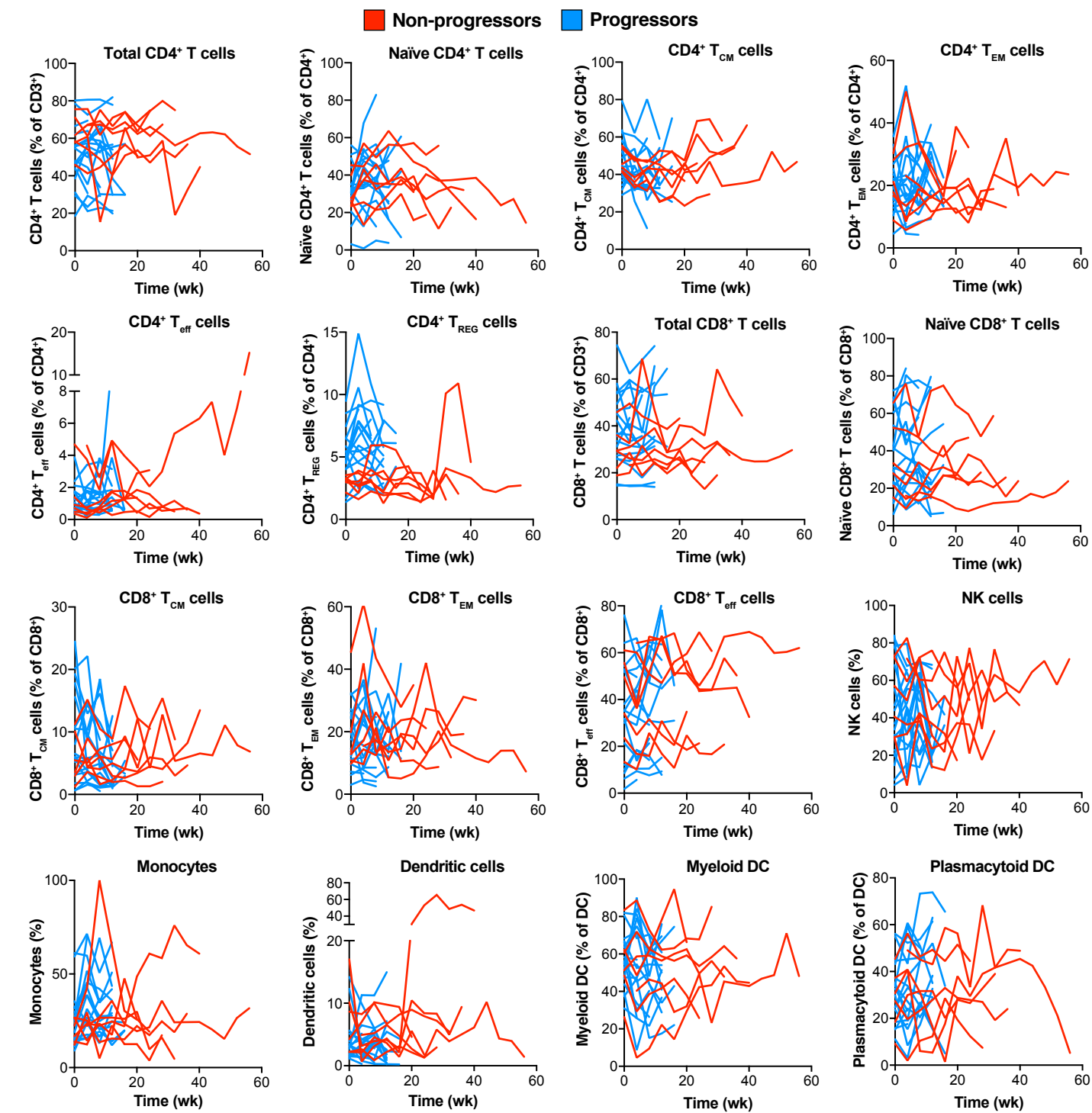

Supplement: Supplementary file 5 — Additional file 5 : Supplementary Figure 5: Longitudinal effects of treatment on leukocyte populations. For each subpopulation, the time-dependent increment or decrement during treatment is shown, using the baseline sample as reference. Red charts: responders; blue charts: non-responders. [file 13058_2020_1362_MOESM5_ESM.pdf]
