# Supplementary material for: Immuno-priming durvalumab with bevacizumab in HER2-negative advanced breast cancer: a pilot clinical trial
Source: Breast Cancer Res. 2020 Nov 11;22:124. doi: 10.1186/s13058-020-01362-y (PMC7661209; doi:10.1186/s13058-020-01362-y)
Supplement: Supplementary file 6 — Additional file 6 : Supplementary Table 1: Antibodies used for immunophenotyping in peripheral blood. [file 13058_2020_1362_MOESM6_ESM.docx]

**Supplementary Tables**

**Supplementary Table 1: Antibodies used for immunophenotyping in peripheral blood**

| **PANEL** | **MARKER** | **CLONE** | **LABEL** | **ISOTYPE** | **PROVIDER** |
| --- | --- | --- | --- | --- | --- |
| T cells | Dead/live | -- | Violet | -- | INVITROGEN |
|  | CD3 | UCHT1 | FITC | IgG1 | BECKMAN |
|  | CD4 | 13B8.2 | PC5.5 | IgG1 | BECKMAN |
|  | CD8 | B9.11 | APCa750 | IgG1 | BECKMAN |
|  | CD45RA | 2H4 | PC7 | IgG1 | BECKMAN |
|  | CCR7 | 150503 | PE | IgG2a | R&D Systems |
|  | CD38 | LS198-4-3 | APC | IgG1 | BECKMAN |
|  | HLA-DR | Immu-357 | KrO | IgG1 | BECKMAN |
| Treg  cells | Dead/live | -- | Violet | -- | INVITROGEN |
|  | CD3 | UCHT1 | FITC | IgG1 | BECKMAN |
|  | CD4 | 13B8.2 | PC5.5 | IgG1 | BECKMAN |
|  | CD25 | 1HT44H3 | APCa750 | IgG2a | BECKMAN |
|  | CD45RO | UCHL1 | PC7 | IgG1 | BECKMAN |
|  | CCR4 | 205410 | PE | IgG2b | R&D Systems |
|  | CD127 | R34.34 | APC | IgG1 | BECKMAN |
|  | HLA-DR | Immu-357 | KrO | IgG1 | BECKMAN |
| NK cells  Monocytes  Dendritic cells | Dead/live | -- | Violet | -- | INVITROGEN |
|  | CD3 | UCHT1 | FITC | IgG1 | BECKMAN |
|  | CD19 | J3-119 | FITC | IgG1 | BECKMAN |
|  | CD20 | B9E9 (HRC20) | FITC | IgG2a | BECKMAN |
|  | CD123 | SSDCLY107D2 | PC5.5 | IgG1 | BECKMAN |
|  | CD14 | RMO52 | APCa750 | IgG2a | BECKMAN |
|  | CD11c | BU15 | PC7 | IgG1 | BECKMAN |
|  | CD56 | N901 (NKH.1) | PE | IgG1 | BECKMAN |
|  | CD16 | 3G8 | APC | IgG1 | BECKMAN |
|  | HLA-DR | Immu-357 | KrO | IgG1 | BECKMAN |
